# Influencing Factors of Hexavalent Chromium Speciation Transformation in Soil from a Northern China Chromium Slag Site

**DOI:** 10.3390/molecules30153076

**Published:** 2025-07-23

**Authors:** Shuai Zhu, Junru Chen, Yun Zhu, Baoke Zhang, Jing Jia, Meng Pan, Zhipeng Yang, Jianhua Cao, Yating Shen

**Affiliations:** 1 Key Laboratory of Eco-Geochemistry, Ministry of Natural Resources of China, National Research Center for Geoanalysis, Beijing 100037, China; zhu15131215153@126.com (S.Z.); chenjunru1234@163.com (J.C.); zhuyun@mail.cgs.gov.cn (Y.Z.); zhangbaoke@mail.cgs.gov.cn (B.Z.); jiajing@mail.cgs.gov.cn (J.J.); spmile3629@163.com (M.P.); zhangzhipeng@mail.cgs.gov.cn (Z.Y.); 2 Institute of Karst Geology, Chinese Academy of Geological Sciences, Guilin 541004, China; cjianhua@mail.cgs.gov.cn; 3 School of Water Resources and Environment, China University of Geosciences (Beijing), Beijing 100083, China

**Keywords:** chromium slag site, hexavalent chromium, contaminated soil, transformation mechanism, remediation strategy, micro-XRF

## Abstract

Chromium slag sites pose severe environmental risks due to hexavalent chromium (Cr(VI)) contamination, characterized by high mobility and toxicity. This study focused on chromium-contaminated soil from a historical chromium slag site in North China, where long-term accumulation of chromate production residues has led to serious Cr(VI) pollution, with Cr(VI) accounting for 13–22% of total chromium and far exceeding national soil risk control standards. To elucidate Cr(VI) transformation mechanisms and elemental linkages, a combined approach of macro-scale condition experiments and micro-scale analysis was employed. Results showed that acidic conditions (pH < 7) significantly enhanced Cr(VI) reduction efficiency by promoting the conversion of CrO_4_^2−^ to HCrO_4_^−^/Cr_2_O_7_^2−^. Among reducing agents, FeSO_4_ exhibited the strongest effect (reduction efficiency >30%), followed by citric acid and fulvic acid. Temperature variations (−20 °C to 30 °C) had minimal impact on Cr(VI) transformation in the 45-day experiment, while soil moisture (20–25%) indirectly facilitated Cr(VI) reduction by enhancing the reduction of agent diffusion and microbial activity, though its effect was weaker than chemical interventions. Soil grain-size composition influenced Cr(VI) distribution unevenly: larger particles (>0.2 mm) in BC-35 and BC-36-4 acted as main Cr(VI) reservoirs due to accumulated Fe-Mn oxides, whereas BC-36-3 showed increased Cr(VI) in smaller particles (<0.074 mm). μ-XRF and correlation analysis revealed strong positive correlations between Cr and Ca, Fe, Mn, Ni (Pearson coefficient > 0.7, *p* < 0.01), attributed to adsorption–reduction coupling on iron-manganese oxide surfaces. In contrast, Cr showed weak correlations with Mg, Al, Si, and K. This study clarifies the complex factors governing Cr(VI) behavior in chromium slag soils, providing a scientific basis for remediation strategies such as pH adjustment (4–6) combined with FeSO_4_ addition to enhance Cr(VI) reduction efficiency.

## 1. Introduction

With the acceleration of industrialization, substantial amounts of chromium-containing pollutants have entered the soil environment [1]. These pollutants are primarily transported through industrial wastewater discharge, waste residue accumulation and atmospheric deposition. Chromium slag, a general term for waste discharged during the production of metallic chromium, chromium salts, and ferrochromium, contains hexavalent chromium (Cr(VI)) as its most toxic component [2]. Due to its carcinogenic, mutagenic, and acute toxic properties, Cr(VI) exerts one of the most significant environmental impacts [2]. It not only severely contaminates surrounding soil environments [3] but also damages soil ecosystems and microbial community structures [4], and easily infiltrates groundwater, posing threats to both the environment and human health [4]. According to the United Nations Environment Programme (UNEP) assessment report, the global annual emission of total chromium from industrial activities exceeds 2.6 × 10^6^ tons, with China contributing more than 38% of this total [5]. To address such issues, countries and regions worldwide have established corresponding regulatory standards for Cr(VI) levels in soil. For instance, calculations based on the risk assessment model specified in the standards of the United States Environmental Protection Agency (USEPA) indicate that the minimum Health-Based Action Levels (HBALs) for soil Cr(VI) in non-residential areas is 105 mg/kg [6]; China’s Soil Environmental Quality: Risk Control Standard for Soil Contamination of Development Land (GB 36600-2018) [7] which stipulates a screening value of 78 mg/kg for Cr(VI) in Type II land. Currently, China has nearly 26 abandoned chromate manufacturers and over 40 chromium slag sites, where soil Cr(VI) concentrations far exceed national environmental standards [8]. Therefore, investigating the migration and transformation mechanisms of Cr(VI) in soil and developing efficient remediation technologies are crucial for controlling pollution risks and ensuring the safe reuse of contaminated land.

The environmental behavior and toxicity of chromium are strongly governed by its chemical speciation. Trivalent chromium (Cr(III) and Cr(VI) are the most common and relatively stable chromium states in soils [9], but their effects on human health and the natural environment differ significantly [10], a speciation-dependent difference that directly drives their distinct impacts. Cr(VI) is highly bio-accumulative in plants and animals, eventually damaging human health through food chain accumulation [11]. Consequently, the International Agency for Research on Cancer (IARC) has classified Cr(VI) as a Group 1 human carcinogen [12]. It accumulates in the liver, kidneys, heart, blood, and endocrine glands via oral ingestion, skin contact, and inhalation, causing adverse liver effects [13] and increased cancer risk [14]—occupational exposure is linked to lung, nasal, and sinus cancer, and suspected to relate to gastric and laryngeal cancers [15]. Critically, Cr(VI) exhibits extremely high solubility and mobility across a broad pH range: under acidic conditions (pH < 6); it is dominated by hydrogen chromate (HCrO_4_^−^) with partial conversion to dichromate (Cr_2_O_7_^2−^); under alkaline conditions (pH > 7), it primarily exists as chromate (CrO_4_^2−^) [16]. This mobility allows rainwater, irrigation, or wastewater to drive continuous release of Cr(VI) leachate into groundwater [8]. In contrast, Cr(III) is generally pharmacologically active, improving glucose metabolism [17] and regulating lipid metabolism [18,19], though high occupational exposure can cause eardrum damage [20]. Geochemically, Cr(III) exists in soil as low-solubility chromium hydroxide (Cr(OH)_3_) precipitates, resulting in low mobility [21].

In complex, heterogeneous soil environments, Cr(VI) speciation is not static. Its valence changes are influenced by soil composition, pH, and organic matter [22,23]. For example, Cr(III) may be oxidized to Cr(VI) under oxidizing and alkaline conditions, while Cr(VI) is easily reduced to Cr(III) under reducing conditions [24]. Reducing agents (e.g., humic acid) can reduce Cr(VI) to less toxic Cr(III) [25]. Additionally, soil involves intricate physical, chemical, and biological interactions between chromium and elements such as iron, manganese, aluminum, and silicon [26], which shape both speciation transformation and microscale elemental distribution [26]. Thus, understanding chromium’s microstructure distribution is key to clarifying its environmental behavior. However, microscale analysis of chromium distribution and multi-element interactions remains challenging. While techniques like SEM–EDS [27,28] and synchrotron radiation [29] enable microscopic elemental studies, they are costly and require strict sample conditions. Further, most research uses artificially homogeneous soils [25], whereas actual chromium slag site soils are highly heterogeneous. Cr(VI) here exists in aged forms, forming complex associations with iron/manganese oxides, clays, or organic matter [30]. This gap between lab simulations and real pollution hinders accurate reflection of natural Cr(VI) occurrence.

Current research on Cr(VI)-contaminated soils focuses on total chromium content, speciation, and extractability via chemical methods [31], but lacks systematic analysis of multi-factor influences, particularly in revealing chromium’s interactions with other elements across micro-to-macro scales. To address this gap, this study focuses on actual chromium slag site soils, adopting a multi-dimensional strategy integrating macro and micro-scale perspectives to explore Cr(VI) valence changes and total Cr distribution rules in real environments. At the macro level, we analyze the effects of pH, organic matter types (e.g., humic acid, fulvic acid), and reducing agent addition on Cr(VI) transformation. At the micro scale, μ-XRF captures spatial distributions of Cr, Fe, Mn, Al, and other elements in soil microstructures to reveal element correlations. This work provides theoretical support for remediating chromium-contaminated soils, optimizes pollution control measures, and promotes safe redevelopment of contaminated sites.

## 2. Results and Discussion

### 2.1. The Pollution Status of Chromium (VI) in the Chromium Slag Site in North China

Three soil samples (designated BC-36-3, BC-36-4, and BC-35) from the chromium slag site were analyzed with 11 replicate determinations. The total chromium contents were 16,484, 15,326, and 15,796 mg/kg, respectively, while the average Cr(VI) contents were 2124, 1926, and 3509 mg/kg in sequence (see Table S1). Cr(VI) accounted for 13–22% of the total chromium content across all samples. Notably, the Cr(VI) concentrations exceeded the screening value of 78 mg/kg for Type II land specified in China’ Soil Environmental Quality: Risk Control Standard for Soil Contamination of Development Land (GB 36600-2018) [7] by 20–40 times, indicating severe Cr(VI) pollution in the studied soils. Leakage of Cr(VI) from contaminated soils typically originates from aqueous CrO_4_^2−^ solutions, such as leachate generated from chromium residue piles during rainfall events. Consequently, Cr(VI) initially enters the soil matrix as water-soluble CrO_4_^2−^, subsequently undergoing complex adsorption and precipitation reactions with soil minerals [30].

### 2.2. Influence of PH on Cr(VI)

**Figure 1:**
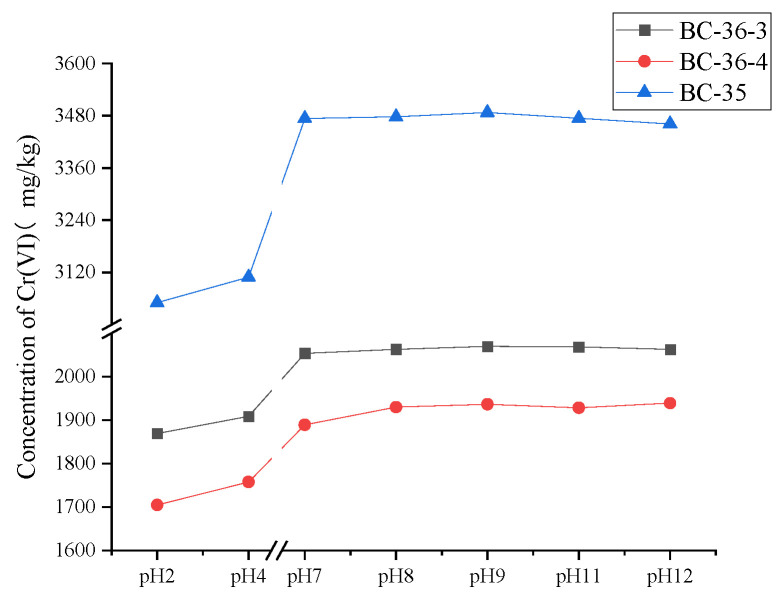
Influence of pH on Cr(VI) content in three chromium slag site soils.

The relationship between Cr(VI) content and pH across the three soil samples is shown in Figure 1. Soil pH is an important parameter governing Cr(VI) speciation and redox ability. It is important to note that all three exhibited alkaline conditions in their initial state, with Cr(VI) primarily existing as CrO_4_^2−^ (chromate ion). Under varying pH conditions, the Cr(VI) content in all three soils displayed a consistent trend: as pH decreased, soil Cr(VI) content decreased, indicating that acidic conditions are more conducive to Cr(VI) reduction. This pH-dependent variation in Cr(VI) content arises from the speciation shifts of Cr(VI) across different pH ranges. Specifically, HCrO_4_^−^ and Cr_2_O_7_^2−^ dominate at pH 0–6; CrO_4_^2−^ emerges at around pH 4.5, reaches maximum abundance at pH ≥ 8, and maintains this state at higher pH values [24].

Cr(VI) acts as a strong oxidant with a high standard redox potential (E^0^(Cr(VI)/Cr(III)) = 1.35 V) in acidic environments [32]. Its stability increases with pH, particularly near neutral and alkaline conditions where the standard redox potential drops to −0.12 V [33]. Since the electrode potential for Cr(VI) reduction to Cr(III) is higher under acidic conditions than alkaline conditions, significant differences in Cr(VI) content were observed at pH < 7, whereas these differences diminished at pH > 7.

When the soil pH value was adjusted to 2, the concentration of Cr(VI) in BC-36-3, BC-36-4 and BC-35 soil samples decreased by 9.42%, 11.42% and 12.22%, respectively, compared with the mean values recorded at pH values 7, 8, 9, 11 and 12. At pH 4, the concentration of Cr(VI) in each soil sample also decreased by 7.50%, 8.67% and 10.52% relative to the mean values at pH 7, 8, 9, 11 and 12.

### 2.3. Influence of Organic Matter on Cr(VI) Behavior

#### 2.3.1. Influence of Fulvic Acid on Cr(VI)

**Figure 2:**
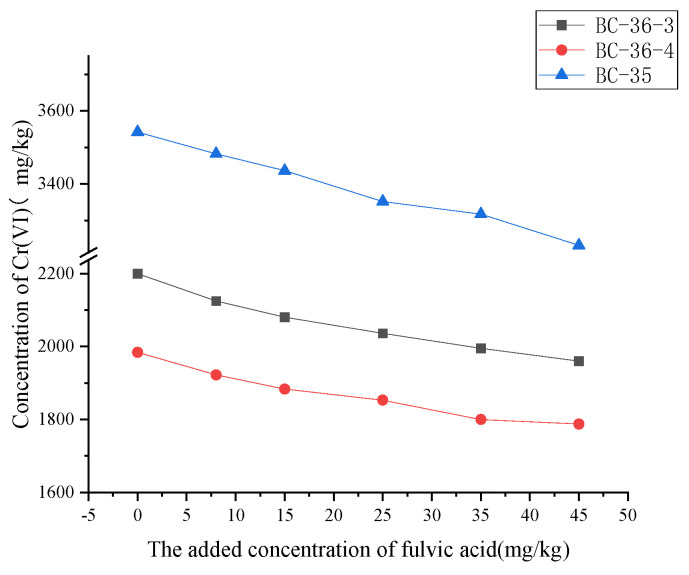
Influence of fulvic acid on Cr(VI) content in three chromium slag site soils.

Changes in Cr(VI) content following adjustments to fulvic acid addition in contaminated soils are presented in Figure 2. Cr(VI) content in contaminated soils gradually decreased with increasing fulvic acid concentration, indicating that fulvic acid exerts a significant reduction effect on soil Cr(VI). At a fulvic acid addition level of 45 mg/kg, the Cr(VI) contents in BC-36-3, BC-36-4, and BC-35 samples decreased by 10.89%, 11.42%, and 12.22%, respectively. As a component of soil humus, fulvic acid’s reduction effect operates through multiple mechanisms:

Firstly, it contains abundant active functional groups (e.g., phenolic hydroxyl and carboxyl groups) that directly reduce Cr(VI) to Cr(III) [34]. Secondly, since Cr(VI) reduction to Cr(III) in soil is closely linked to soil organic carbon content, fulvic acid—an important component of organic carbon—can enhance this reduction reaction by increasing soil organic carbon levels [35]. Fulvic acid can form complexes with soil Fe(II), indirectly accelerating Cr(VI) transformation by enhancing Fe(II)’s reductive activity [36]. Meanwhile, fulvic acid addition significantly increases soil hydrogen ion (H^+^) concentration, lowering soil pH. This acidic environment enhancement provides favorable conditions for Cr(VI) to Cr(III) transformation, consistent with the principle that “soil Cr(VI) reduction to Cr(III) is pH-dependent” [35]. In such conditions, Cr(VI) is more prone to reduction to Cr(III), and these combined effects ultimately reduce soil Cr(VI) content.

#### 2.3.2. Influence of Citric Acid on Cr(VI)

**Figure 3:**
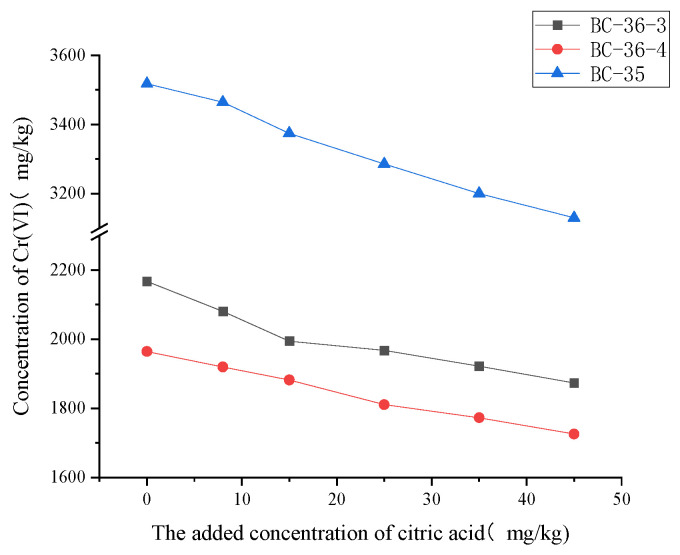
Influence of citric acid on Cr(VI) content in three chromium slag site soils.

Changes in Cr(VI) content following adjustments to citric acid addition in chromium-contaminated soils are shown in Figure 3. Cr(VI) content gradually decreased with increasing citric acid concentration, indicating that citric acid also exerts an obvious reduction effect on soil Cr(VI) through multiple mechanisms.

In direct action, citric acid acts as an electron donor to directly participate in reactions, accelerating Cr(VI) conversion to Cr(III) [37]; additionally, the reduction intermediate Cr(V) readily complexes with citrate, promoting continuous reaction progression [38]. In indirect action, citric acid serves as a microbial carbon source to enhance iron and manganese oxide reduction capacity [39], synergizes with Mn(II) to strengthen the reduction effects [40], and promotes iron oxide dissolution, with released Fe(II) directly reducing Cr(VI) [41]. Meanwhile, citric acid addition lowers soil pH and increases dissolved organic carbon, providing favorable conditions and electron donors for reduction [39]. At a citric acid addition level of 45 mg/kg, Cr(VI) contents in BC-36-3, BC-36-4, and BC-35 samples decreased by 13.56%, 12.16%, and 10.45%, respectively, compared to samples without citric acid addition.

The linear regression equation slope of citric acid was generally larger than that for fulvic acid, suggesting that, at equivalent addition levels, citric acid has a more significant effect on reducing Cr(VI) concentration, indicating slightly stronger Cr(VI) reduction capacity than fulvic acid, likely due to its simpler molecular structure. Once Cr(VI) is reduced to Cr(III), it reacts with citric acid’s carboxyl groups (−COOH) to form stable complexes. This complex formation improves chromium stability in soil and effectively inhibits Cr(VI) re-release [42], thereby reducing chromium bioavailability and toxicity.

### 2.4. Influence of FeSO_4_ on Cr(VI)

**Figure 4:**
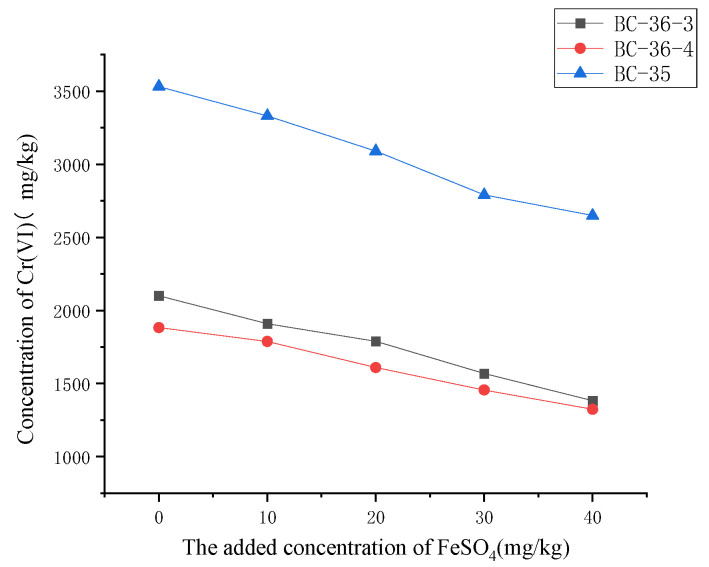
Influence of FeSO_4_ on Cr(VI) content in three chromium slag site soils.

Fe(II)-mediated reduction of Cr(VI) mainly occurs in anaerobic environments. In the presence of O_2_, Fe(III) becomes the dominant species (as Fe(II) is rapidly oxidized to Fe(III)), and Fe(III) cannot reduce Cr(VI). Therefore, after adjusting the FeSO_4_ addition amount in contaminated soils, samples were sealed and stored to maintain anaerobic conditions, with results shown in Figure 4. As FeSO_4_ content increased, Cr(VI) content in contaminated soils gradually decreased, indicating that FeSO_4_ exerts a significant reduction effect on soil Cr(VI). Iron plays a dual role in this process: electron donation (through Fe^2+^ oxidation to Fe^3+^) and adsorption via precipitation/adsorption mechanisms. The standard electrode potential for the Fe^3+^/Fe^2+^ couple is relatively low at 0.77 V [33], facilitating electron transfer to Cr(VI). Beyond direct Cr(VI) reduction, Fe^2+^ can also act as an electron transport medium to accelerate the reduction reaction between Fe^0^ and Cr(VI) [37]. Notably, FeSO_4_ exhibits stronger Cr(VI) reduction capacity than fulvic acid and citric acid. Additionally, literature reports indicate that Cr(VI) reduction by Fe(II) is a relatively rapid process (occurring within tens of seconds to several hours), faster than reduction by organic matter [33].

**Figure 5:**
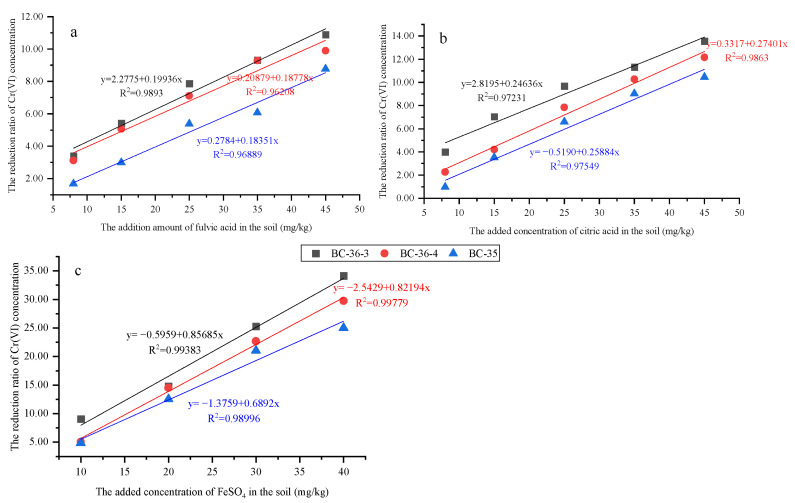
Effect of fulvic acid (**a**), citric acid (**b**) and FeSO_4_ (**c**) on Cr(VI) reduction in three chromium slag site soils.

As shown in Figure 5, with increasing additions of fulvic acid, citric acid, and FeSO_4_, the Cr(VI) reduction ratios in all three soils exhibited an upward trend, though the magnitude of increase varied. Among the three additives, FeSO_4_ most significantly improved the Cr(VI) reduction ratio, with its fitted linear slope much higher than those of fulvic acid and citric acid. This confirms that FeSO_4_ has strong reducing capacity for Cr(VI) in chromium slag site soils, consistent with literature reports that ferrous sulfate exhibits better reducing effects than humic acid [42]. As a commonly used reducing agent, ferrous sulfate (FeSO_4_) functions primarily by providing ferrous ions (Fe^2+^) for efficient Cr(VI) reduction [43]. This reaction follows first-order kinetics [44], with a rapid reduction rate and high efficiency [45]. Meanwhile, the reduction product Cr(III) and oxidation product Fe(III) form mixed Fe(III)/Cr(III) hydroxide precipitates. These precipitates possess good chemical stability, immobilizing Cr(III) in soil and significantly reducing its mobility and oxidizability [46], thereby further enhancing Cr(VI) transformation and stabilization.

Citric acid exhibited the second strongest reduction effect, with notable increases in Cr(VI) reduction ratios across different soils, indicating its ability to promote Cr(VI) reduction to a certain extent. Its mechanism involves direct participation in Cr(VI) reduction as an electron donor; additionally, complexation between the reduction intermediate Cr(V) and citrate further drives continuous reaction progression [37,38]. Fulvic acid showed the weakest reduction effect but still increased Cr(VI) reduction ratios with higher additions. Fulvic acid may directly reduce Cr(VI) through functional groups like phenolic hydroxyl and carboxyl groups, though with lower reaction efficiency [34], resulting in less significant effects compared to ferrous sulfate and citric acid.

Furthermore, soils with different initial Cr(VI) concentrations showed similar response trends to the three additives but varying specific reduction effects. BC-35 soil, with the highest initial Cr(VI) concentration, exhibited a relatively low reduction ratio at equivalent additive amounts. This may be attributed to intense competitive adsorption: high Cr(VI) concentrations rapidly occupy limited active sites on reducing agents due to their abundance, leading to reductant saturation. This saturation not only hinders further adsorption and reduction of Cr(VI) but may also cause permanent inactivation of active sites if Cr(VI) forms stable complexes with them [47], preventing effective reduction even with residual Cr(VI) in solution. Additionally, Cr(III)—the final product of Cr(VI) reduction—may form insoluble precipitates on reductant surfaces, further blocking contact between Cr(VI) and reducing agents, preventing effective reduction even with residual Cr(VI) in solution. Additionally, Cr(III)—the final product of Cr(VI) reduction—may form insoluble precipitates on reductant surfaces, further blocking contact between Cr(VI) and reducing agents [48] and exacerbating efficiency decline. In contrast, BC-36-3 soil, with intermediate initial Cr concentration, showed the best reduction effect, primarily because reductants could react effectively with Cr(VI) within this concentration range to achieve optimal reduction efficiency.

### 2.5. Influence of Temperature on Cr(VI)

**Figure 6:**
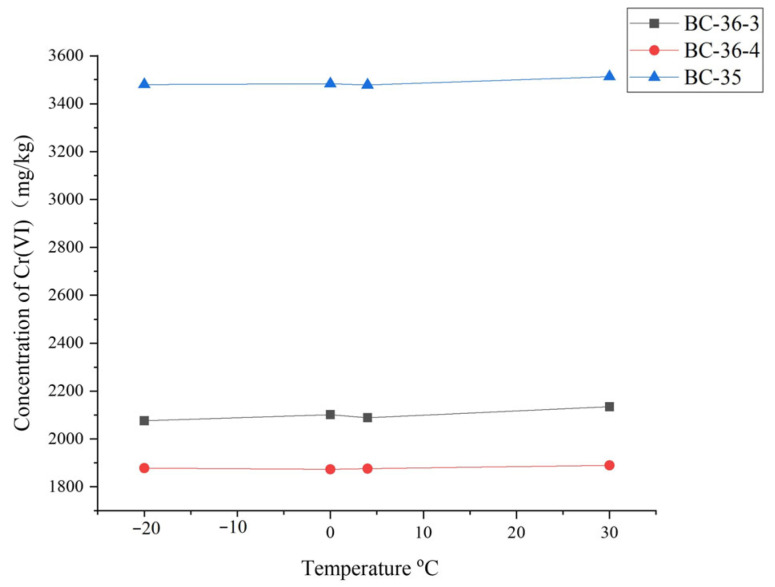
Influence of temperature on Cr(VI) content in three chromium slag site soils.

Changes in Cr(VI) content following temperature adjustments in contaminated soils are shown in Figure 6. Cr(VI) content remained essentially unchanged with temperature variations, with relative standard deviations (RSD) of Cr(VI) measurements in BC-36-4, BC-36-3, and BC-35 soils at –20 °C, 0 °C, 4 °C, and 30 °C being 0.43%, 1.20%, and 1.21%, respectively. These results indicate that temperature had little effect on Cr(VI) transformation in soil within the –20 °C to 30 °C range tested.

While this experiment showed no significant temperature impact on soil Cr(VI) chromium content, microorganisms play a pivotal role in the bio-reductive processes of Cr(VI). Temperature directly influences microbial metabolic activity and enzyme function, thereby regulating microbial abundance, activity, and community structure—factors closely linked to Cr(VI) bio-reduction. Microorganisms employ diverse mechanisms for Cr(VI) reduction, primarily including biosorption, bio-reduction, and biomineralization, which collectively drive Cr(VI) transformation [49]. Temperature changes can significantly affect microbial growth and metabolism, indirectly impacting Cr(VI) reduction efficiency: microbial activity is inhibited above 45 °C, while metabolic rates accelerate with increasing temperature between 0 °C and 35 °C, potentially accelerating organic residue decomposition and promoting Cr(VI) reduction. Additionally, temperature affects microbial adsorption characteristics in soil; for example, parameters in the Langmuir adsorption isotherm model—such as saturated adsorption capacity (*q*ₘ) and equilibrium constant (*K*)—are temperature-dependent [50]. The lack of observed temperature effect in this study may be attributed to the 45-day experimental duration, which may have been insufficient to capture long-term temperature impacts. Some microorganism-mediated Cr(VI) reduction processes require extended periods, as certain microbes must first adapt to the toxic environment before initiating effective reduction [51], making short-term temperature effects less apparent. Alternatively, the soil may have contained insufficient concentrations of organic compounds capable of chelating or reducing Cr(VI) to trigger measurable temperature-dependent reductions.

### 2.6. Influence of Soil Moisture on Cr(VI)

**Figure 7:**
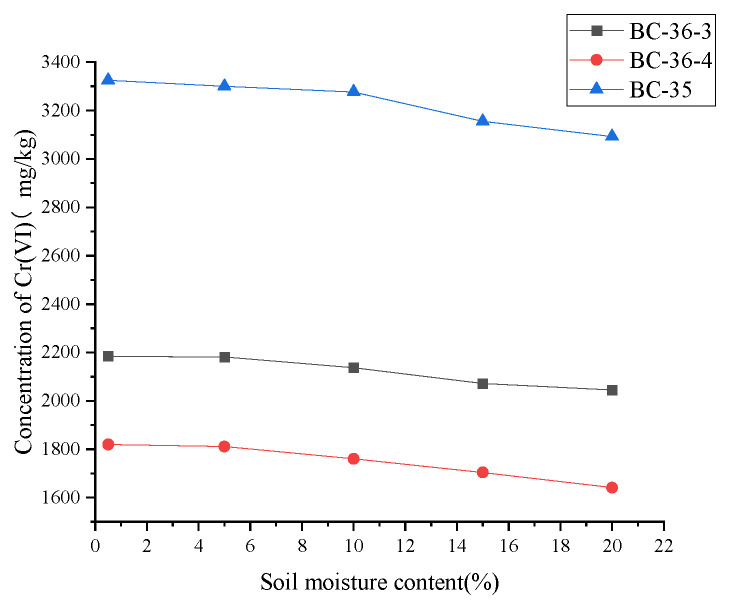
Influence of soil moisture on Cr(VI) content in three chromium slag site soils.

Soil moisture is a key physical property influencing biogeochemical processes. In this study, soil samples were oven-dried at 35 °C to constant weight, then rehydrated with ultrapure water to achieve different moisture contents. While Cr(III) and Cr(VI) speciation in soil depends on multiple factors, soil moisture—alongside soil properties—plays a critical role in Cr(VI) reduction [52]. Changes in Cr(VI) content with soil moisture adjustments are shown in Figure 7. Cr(VI) content gradually decreased as soil moisture increased, indicating that moisture affects soil Cr(VI) dynamics. This moisture-dependent trend may arise because higher soil moisture creates a more favorable medium for Cr(VI) reduction reactions. For example, moisture promotes the dissolution and diffusion of reducing agents, enhancing their contact and reaction with Cr(VI) to increase reduction ratios. When air-dried soil is moistened and extracted in alkaline solution, iron-containing minerals dissolve; Fe(III) can be reduced to Fe(II) by humic substances, which then participates in redox cycles with Cr(VI) as an electron donor. Guidotti et al. [53] reported Fe(II)-mediated Cr(VI) reduction across pH 2–10 and confirmed correlations between Cr(VI) reduction efficiency and iron presence in soil extracts. Additionally, increasing soil moisture gradually restores microbial metabolic activity—microbes play a key role in biological Cr(VI) reduction by converting Cr(VI) to Cr(III) through metabolic processes [54], with enhanced microbial activity under humid conditions accelerating this reduction. Compared to organic matter (fulvic acid, citric acid) and FeSO_4_, moisture exerted a weaker effect on soil Cr(VI). This is because moisture neither directly reacts with Cr(VI) chromium nor provides electron donors like fulvic acid, citric acid, or FeSO_4_—substances that drive reduction through electron transfer. While increased moisture benefits microbial growth and metabolism (and some microbes possess Cr(VI) reduction capabilities), this effect is relatively slow, limited, and constrained by other soil factors (e.g., microbial community composition and nutrient availability).

### 2.7. Influence of Soil Grain-Size Composition on Cr(VI)

**Figure 8:**
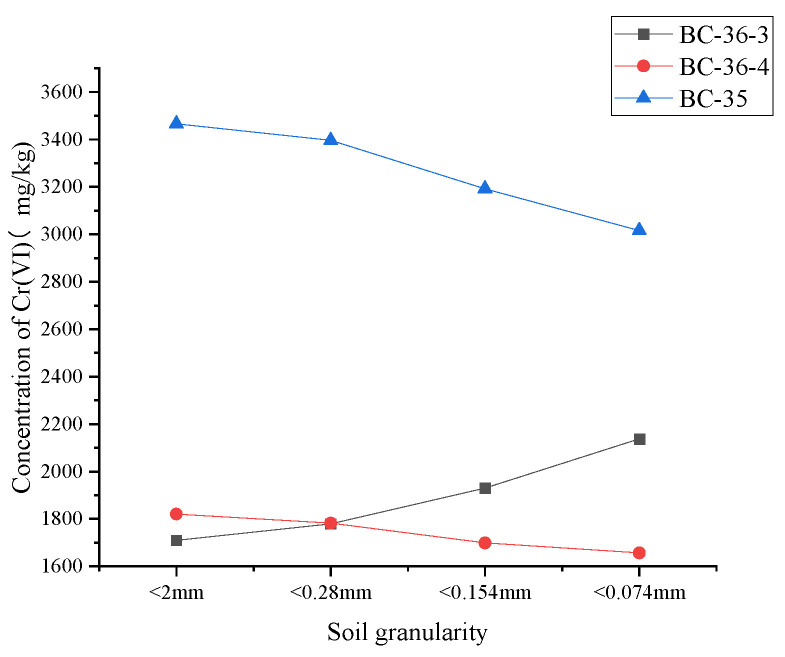
Influence of soil particle size on Cr(VI) content in three chromium slag site soils.

The soil samples were roughly ground and sieved through standard soil sieves of 10, 60, 100, and 200 mesh, respectively. Sieved samples of different particle sizes were collected into soil sample bottles for Cr(VI) content determination. As shown in Figure 8, the Cr(VI) content in the three soil samples exhibited distinct trends with changing particle size. For the BC-35 sample, when particle size was <2 mm, Cr(VI) content was highest at 3466 mg/kg; as particle size gradually decreased to <0.074 mm, Cr(VI) content showed a downward trend, finally dropping to approximately 3016 mg/kg. BC-36-4 exhibited a similar trend to BC-35, with Cr(VI) content gradually decreasing as soil particle size decreased. In contrast, BC-36-3 showed the opposite trend: when soil particle size was <2 mm, Cr(VI) content was lowest at 1710 mg/kg; as particle size continued to decrease, Cr(VI) content gradually increased, reaching approximately 2137 mg/kg at <0.074 mm. This indicates that Cr(VI) content in BC-36-3 was relatively high in smaller particles, with content increasing as particle size decreased.

Generally, coarse sand particles have limited Cr(VI) adsorption capacity due to their relatively small specific surface area, making it difficult for Cr to be retained in coarse sand layers through adsorption [55]. Zhang et al. [56] also found that smaller soil particles, with larger specific surface areas, exhibit stronger Cr(VI) adsorption capacity and thus higher Cr(VI) content. However, the present study showed that Cr(VI) content in coarse sandy fractions of BC-36-4 and BC-35 was significantly higher than in fine-grained fractions. This discrepancy is speculated to arise from differences in mineral composition between coarse and fine particles, leading to varying Cr(VI) fixation and reduction effects.

**Figure 9:**
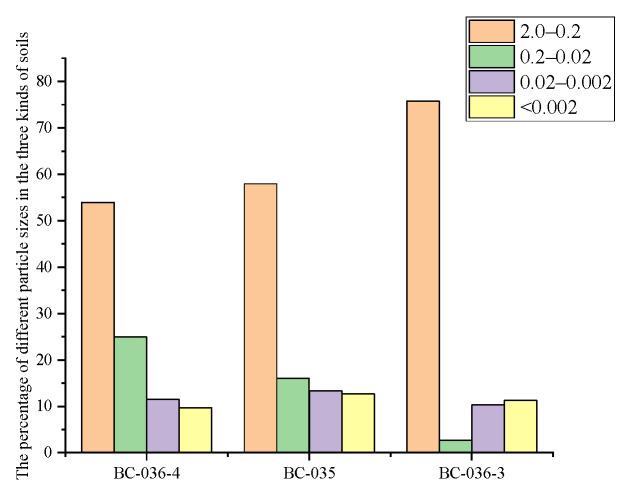
Particle size distribution in three chromium slag site soils.

According to the United States Department of Agriculture (USDA) soil particle classification standard [57], soil particles are categorized into coarse sand (2.0–0.2 mm), fine sand (0.2–0.02 mm), silt (0.02–0.002 mm), and clay (<0.002 mm). In this study, the grain-size composition of the three soils was determined with reference to the China’s industrial standard Soil Testing Part 3: Method for Determination of Soil Mechanical Composition (NY/T 1121.3-2006) [58], and their particle size distributions were plotted in Figure 9. As shown, all three soils are typical sandy loams, with the mass fraction of particles >0.20 mm ranging from 53% to 76%. Particle size distribution in all three soils was dominated by large particles (2.0–0.2 mm), but the proportion of large particles in BC-36-3 was significantly higher than in BC-36-4 and BC-35. Conversely, the proportion of medium particles (0.2–0.02 mm) in BC-36-3 was significantly lower than in the other two soils. The proportion of very fine particles (<0.002 mm) was low in all three soils, with no significant differences. The grain-size composition of BC-36-4 and BC-35 was similar, with fractions in the order of fine sand (0.2–0.02 mm) > silt (0.02–0.002 mm) > clay (<0.002 mm), while BC-36-3 showed the opposite order.

**Figure 10:**
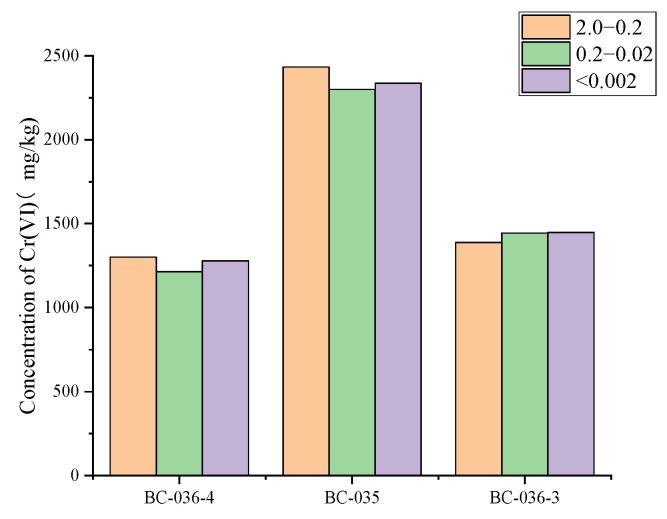
Cr(VI) concentrations in size-fractionated soils from a chromium slag site.

Subsequently, Cr(VI) content in separated soil fractions of different particle sizes was measured, with results plotted in Figure 10. Note that, since silt (0.02–0.002 mm) is included in grain-size composition determinations via wet methods, Figure 10 does not include Cr(VI) content in the silt fraction. As shown, Cr(VI) concentrations in the coarse sand (2–0.2 mm) fraction of BC-36-4 and BC-35 were higher than in fine sand (0.2–0.02 mm) and clay (<0.002 mm) fractions. Combined with Figure 9, which shows coarse sand (2–0.2 mm) as the dominant fraction, this indicates that the 2.0–0.2 mm particle size range is the main storage pool for Cr(VI) in these soils and contributes most to total soil Cr(VI). BC-36-3 showed the opposite pattern: Cr(VI) concentration in coarse sand was lowest (approximately 1388 mg/kg), but this fraction accounted for >75% of the soil mass. Despite its relatively low Cr(VI) content, the large proportion of this fraction makes it the main source of Cr(VI) in BC-36-3.

These results explain the earlier trends: in BC-36-4 and BC-35, Cr(VI) content gradually decreased with decreasing particle size while, in BC-36-3, Cr(VI) content gradually increased with decreasing particle size. X-ray fluorescence spectroscopy (XRF) analysis showed that iron content in coarse particles of BC-35 and BC-36-4 was 7.45% and 9.32%, respectively, significantly higher than in fine particles (6.41% and 8.73%, respectively). In contrast, BC-36-3 showed the opposite trend: iron content in coarse particles (7.63%) was slightly lower than in fine particles (7.89%). This significant difference in iron content between coarse and fine particles is likely a key factor contributing to the relatively high Cr(VI) content in coarse sand fractions. Related studies [59] have noted that iron-containing minerals (e.g., magnetite) can inhibit Cr(VI) reduction reactions, thereby influencing Cr(VI) content distribution in soil.

### 2.8. Correlation Between Chromium and Other Elements

**Figure 11:**
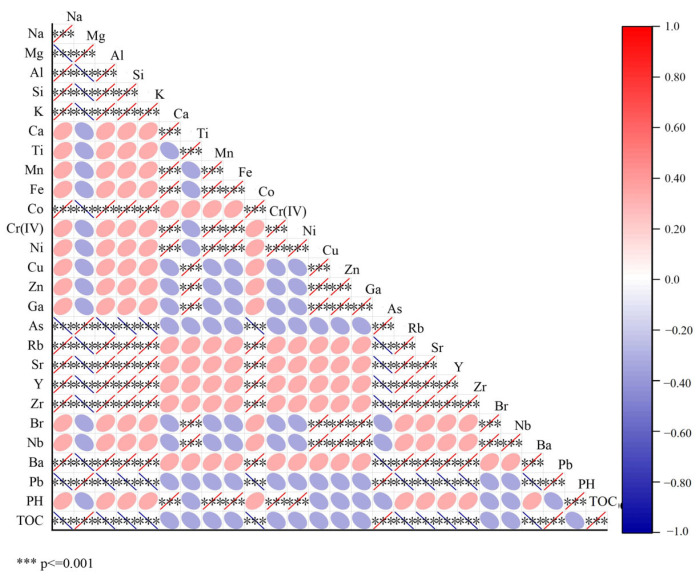
Heat map of the correlations between Cr(VI) and other elements.

**Figure 12:**
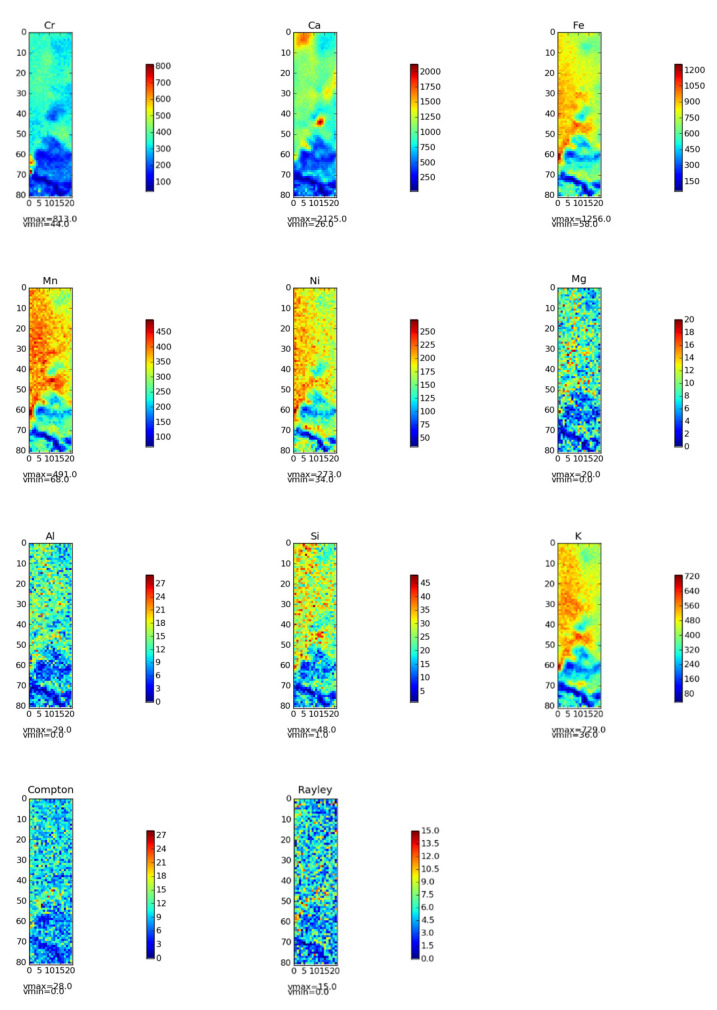
In situ distribution maps of soil elements in a soil profile (depth = 6000 μm, width = 1500 μm) collected from a chromium slag site. Note: In the legend, the color intensity reflects the detection response intensity of the element. Redder colors indicate stronger detection responses in the corresponding area; bluer colors indicate weaker responses.

As shown in Figure 11, correlation analysis was conducted between the content of Cr(VI) in the soil and the contents of other elements. Meanwhile, distribution maps of various elements were generated using micro-XRF (Figure 12). It can be clearly observed from Figure 12 that the distribution of Cr(VI) was remarkably similar to that of Ca, Fe, Mn, and Ni. In different regions of the chromium slag site soil, positions with higher Cr(VI) content tended to have relatively higher Ca, Fe, Mn, and Ni contents. This finding is highly consistent with literature reports: previous studies have confirmed that the spatial coincidence rate between Cr hotspots and soil Fe-Mn nodules exceeds 80%, revealing a strong affinity between Cr and Fe-Mn oxide minerals [60]. This indicates that, in the chromium slag site soil environment, Cr(VI) is not uniformly distributed in soil particles but closely interacts with minerals containing Ca, Fe, Mn, and Ni. Cr(VI) showed positive correlations with Ca, Fe, Mn, and Ni, with some reaching significant levels, which further confirmed the results of the in situ micro-zone distribution map. This indicates that, in chromium slag site soil, Cr(VI) and these elements are not only closely related in spatial distribution but also have significant positive correlations in content.

This relationship may stem from paragenetic associations or coprecipitation between Cr(VI)-bearing minerals and minerals containing Ca, Fe, Mn, and Ni. For example, some iron and manganese oxide minerals have large specific surface areas and abundant surface active sites, which can adsorb or coprecipitate Cr(VI) [61]. At the same time, these minerals may naturally contain Ca, Mn, and Ni, resulting in their distribution in the soil showing a high degree of consistency [62]. Another possible mechanism is redox coupling: Fe and Mn are important redox-active elements in soil, and changes in their oxidation-reduction states affect soil redox potential. Since the stability and mobility of Cr(VI) are closely related to the soil redox environment, redox coupling may occur between Cr(VI) and Fe, Mn, etc., in chromium slag site soil. Specifically, the redox processes of Fe and Mn affect the reduction or oxidation of Cr(VI), leading to strong correlations in their soil distribution. The specific mechanisms are as follows: The redox processes of iron and manganese are closely linked to chromium speciation transformation. Manganese oxides in soil, especially easily reducible ones, are closely associated with the oxidation of Cr(III) to Cr(VI) and can directly oxidize Cr(III) to form Cr(VI) [63]. Additionally, the manganese redox cycle affects the fate, migration, and distribution of chromium [64]. In contrast, Fe(II) influences chromium speciation through two opposing mechanisms: on the one hand, Fe(II) can directly reduce Cr(VI) to Cr(III), thereby reducing environmental risks [65]; on the other hand, reducible soluble iron (Fe(II)_a_q) can cause passivation of manganese oxide surfaces, weakening their ability to oxidize Cr(III) [66] and further regulating chromium speciation distribution. Ultimately, these interactions result in similar distribution patterns of iron, manganese, and chromium in soil.

In contrast, Cr(VI) showed varying degrees of weak association with Mg, Al, Si, and K. The in situ micro-zone distribution map revealed significant differences in distribution between Cr(VI) and Mg, Al, Si, K, with no obvious similarity. Meanwhile, the correlation heatmap showed weak or even negative correlations between Cr(VI) and Mg, Al, Si, and K, none of which reached significant levels. This mutual confirmation with the in situ micro-zone distribution results indicates that Cr(VI) has no obvious correlation with these elements in chromium slag site soil. Cr(VI) exhibits strong oxidizing property and high mobility, and is easily affected by soil redox conditions, pH, and other factors, leading to migration and transformation. In contrast, elements such as Mg, Al, Si, and K exhibit relatively stable chemical behavior in soil, mainly participating in soil structure formation and ion exchange processes [67]. Their mobility and chemical activity are significantly different from those of Cr(VI), resulting in no obvious correlation in soil distribution.

## 3. Materials and Methods

### 3.1. Soil Sample Collection

This study was conducted at a historical chromium-contaminated site in North China. Historically, this site was mainly engaged in sodium dichromate production, with chromium slag generated during production accumulated on-site for a long time. Chromium slag originates from core chromate production processes (oxidation roasting and alkaline leaching): chromite ore and sodium carbonate are roasted at high temperatures to produce soluble chromates; after target products are extracted via alkaline leaching, remaining insoluble residues form chromium slag, which contains unreacted minerals, iron-manganese oxides, residual Cr(VI), etc. Among these, Cr(VI) has become the main source of soil pollution risk due to its high mobility and biological toxicity.

The soil at this chromium slag site is alkaline, with a pH range of 8.16–9.37. This alkalinity is attributed to chromate production processes involving oxidation roasting and alkaline leaching [55]. Soil organic matter content at the site was low (only 2.55%), possibly due to inhibition of soil microbial growth and activity by potentially toxic elements (PTEs) such as chromium, which prevents organic matter decomposition, transformation, and accumulation. Additionally, the alkaline environment of chromium slag alters soil pH, which is unfavorable for organic matter accumulation.

Soil samples were collected from 0–10 cm topsoil and 30–40 cm deep soil to cover gradients of Cr(VI) concentration and Cr(VI)/total chromium ratio, supporting subsequent macro–micro mechanism studies. Among them, BC-36-3 and BC-35 (both 0–10 cm topsoil) represent spatial heterogeneity across the site, while BC-36-4 (30–40 cm deep soil) corresponds vertically to BC-36-3, enabling analysis of vertical distribution characteristics. Selected samples have Cr(VI) contents ranging from 1926 to 3509 mg/kg and Cr(VI)/total Cr ratios of 13–22%, showing obvious gradient differences to support comparative analysis of Cr(VI) transformation patterns under different pollution levels. Each sample group had a total mass of approximately 1000 g, with sampling conducted using non-metallic tools (plastic or wooden shovels) to avoid cross-contamination.

Collected soil samples were air-dried naturally to maintain the stability of soil mineral structure and Cr(VI) occurrence forms. Subsequently, impurities such as litter and stones were removed via 10-mesh sieving to ensure matrix purity. After coarse sieving, soil clods were ground using a ball mill and passed through a 100-mesh sieve to prepare uniform fine soil samples, effectively reducing particle effect interference. Finally, sieved samples were sealed in sterile containers and stored in the dark to isolate influences of external factors (e.g., humidity, oxidation conditions) on Cr(VI) forms.

A 0–20 cm deep soil profile was collected at the North China chromium slag site to systematically reflect the vertical distribution characteristics of chromium pollution. During collection, a stainless steel sampling tube was used for integral sampling to avoid profile layer disturbance. After collection, samples were immediately wrapped in plastic wrap and fixed in foam-filled sampling boxes to prevent extrusion and scattering during transportation, ensuring sample integrity. Upon arrival at the laboratory, samples were air-dried in a ventilated, cool location (avoiding direct sunlight) to prevent changes in Cr(VI) forms. After drying to appropriate hardness, relatively flat vertical profiles were selected for in situ micro-X-ray fluorescence (μ-XRF) measurement, providing microscale visual data support for analyzing vertical migration and transformation mechanisms of chromium in soil.

### 3.2. Instruments and Reagents

Inductively coupled plasma optical emission spectrometer (ICP-OES, Avio^®^500, PerkinElmer, Waltham, MA, USA). Water bath oscillator (SHA-B, Lichen Instrument Technology Co., Ltd., Shanghai, China). JX-2GL planetary ball mill (Jingxin Co., Ltd., Shanghai, China). High-speed centrifuge (HC-3018R, Zhongke Zhongjia Scientific Instrument Co., Ltd., Hefei, Anhui, China). pH meter (FiveEasy Plus, Mettler Toledo International Inc., Medina, OH, USA). TOC analyzer (Analytik Jena AG, Jena, Germany). X-ray fluorescence spectrometer (PANalytical, Almelo, Netherlands). Electric ball mill (Retsch GmbH, Haan, Germany) with an oscillation frequency of 30 Hz (or 30 times per second), equipped with 28 mL mixing bottles and 10 mm agate balls. Tablet press (Maekawa Testing Machine Mfg Co., Ltd. Tokyo, Japan) with a maximum pressure of 480 MPa, equipped with a 36 mm sample pressing mold. situ micro-area X-ray fluorescence spectrometer (National Research Center for Geoanalysis, Beijing, China).

Sodium hydroxide (NaOH, AR), dipotassium hydrogen phosphate (K_2_HPO_4_, AR), potassium dihydrogen phosphate (KH_2_PO_4_, AR), anhydrous sodium carbonate (Na_2_CO_3_, AR), hydrochloric acid (HCl, AR), citric acid (C_6_H_8_O_7_, AR), and ferrous sulfate (FeSO_4_·7H_2_O, AR) were all purchased from Sinopharm Chemical Reagent Co., Ltd., China; anhydrous magnesium chloride (MgCl_2_, analytical grade) was purchased from Aladdin Biochemical Technology Co., Ltd., Shanghai, China; fulvic acid (C_14_H_12_O_8_, 95%) was purchased from Macklin Biochemical Technology Co., Ltd., Shanghai, China Ultrapure water was used as the experimental water.

### 3.3. Determination of Basic Soil Properties

Soil pH was measured using a pH meter at a soil/water ratio of 2:5 (g/mL). Total organic carbon (TOC) content was determined using a TOC analyzer.

Cr (VI) was determined via alkali extraction combined with ICP-OES, modified from China’s Soil and Sediment—Determination of Chromium(VI)—Alkaline Digestion/Flame Atomic Absorption Spectrometry (HJ1082-2019) [68]: 0.5 g soil sample was accurately weighed and transferred to a 250 mL volumetric flask; 10.0 mL alkaline extraction solution (30 g/L Na_2_CO_3_ + 20 g/L NaOH), 1 mL MgCl_2_ solution (80 mg/mL), and 0.5 mL buffer solution (17.42 mg/mL K_2_HPO_4_ + 13.6 mg/mL KH_2_PO_4_) were added sequentially. The mixture was heated in a 94 °C water bath with oscillation at 240 r/min. This operation was conducted in an alkaline environment (pH > 11.5), where soluble trivalent chromium (Cr(III)) forms chromium hydroxide precipitate (Cr(OH)_3_). Simultaneously, synergistic effects of magnesium chloride and phosphate buffer effectively suppressed interference from trivalent chromium during Cr(VI) determination. After cooling to room temperature, the extract was filtered into a 100 mL volumetric flask, pH adjusted to weakly acidic using concentrated nitric acid, volume made up to the mark, and finally analyzed by ICP-OES, method reliability was confirmed using the certified reference material RMH-A043, with relative errors <6% and RSD <5% (Table S2), ensuring accurate quantification of Cr(VI).

Total chromium and mineral contents (Fe, Mn, K, etc.) were determined as follows: 7.0000 g sample and 1.5750 g paraffin powder were precisely weighed into a 28 mL mixing bottle with two 10 mm agate balls. The mixture was homogenized using an electric ball mill at 30 Hz for 2 min, then pressed into 36 mm diameter circular pellets under 20 MPa pressure. Sample numbers were marked on pellet backs for X-ray fluorescence spectrometer analysis, with instrument power controlled at ≤3630 W. Detailed element-specific analytical conditions are provided in the relevant literature [69].

### 3.4. Soil Element Distribution Determination

To investigate distribution patterns of Cr(VI) and other potentially toxic elements (PTEs) in soil, non-destructive two-dimensional elemental scanning was performed using an in situ micro-area X-ray fluorescence spectrometer. The instrument was equipped with a focusing capillary lens as the microbeam X-ray source, a rhodium anode target [70], and had a focal spot size of ~30 μm × 30 μm. Silicon drift detectors with a resolution of 130 eV at 5.9 keV (FWHM) were used, with the X-ray tube positioned at 45° to the detector and mounted above a 3D-controlled sample stage. Excitation voltage was 48 kV and current 0.8 mA. Before analysis, instrument energy calibration was performed using copper foil. Soil samples were scanned for 300 s to identify interference-free characteristic spectral lines [71,72,73], and regions of interest corresponding to characteristic peaks were outlined. A 6000 μm × 1500 μm area of the soil profile was scanned with 100 μm horizontal and vertical step sizes, with 5 s of analysis per scanning point.

### 3.5. Setting of Experimental Conditions for Controlled Experiments

To explore the effects of various factors on soil Cr(VI) content, experiments were conducted under different conditions of pH, organic matter (fulvic acid, citric acid) addition, FeSO_4_ addition, temperature, humidity, and soil particle size. Each experiment lasted 45 days with 3 replicates to reduce errors, and samples were incubated in a 25 °C constant temperature incubator in the dark (except temperature experiments).

pH influence experiment: Three contaminated soil samples were selected, and the pH value of the soil was adjusted to different levels, such as 2, 4, 6, 8, 9, 11 and 12, respectively by adding acid-base regulator, and the content of Cr(VI) in the soil under each pH value was determined.

Organic matter influence experiment: Different amounts of fulvic acid and citric acid were added to the three selected polluted soils, respectively (fulvic acid and citric acid are first prepared into 10 g/L stock solutions), the additional amounts were set to 0, 15, 30, 45 mg/kg, etc., and the changes in the content of Cr(VI) in the soil were measured after the addition.

FeSO_4_ influence experiment: Various amounts of FeSO_4_ were added to the three selected contaminated soils (FeSO_4_ is prepared into a 20 g/L stock solution, which is prepared and used immediately to avoid the oxidation of Fe^2+^). The addition gradients were 0, 100, 200, 300 mg/kg, etc. Since Fe(II) reduction of Cr(VI) mainly occurred in anaerobic environments, the samples were adjusted and sealed. The changes in the content of Cr(VI) in the soil were measured.

Temperature influence experiment: Three selected contaminated soils were cultured at −20 °C, 0 °C, 4 °C and 30 °C for 45 days, respectively, to determine the content of Cr(VI) in the soil, and analyze the influence of temperature on Cr(VI) transformation.

Humidity influence experiment: The three selected contaminated soils were first heated in a low-temperature (35 °C) drying oven until completely dry, and then different amounts of ultra-pure water were added to prepare soil samples with moisture contents of 0.5%, 10%, 15%, and 20%. The content of Cr(VI) in soil was determined after being air-dried in the laboratory under different humidity conditions.

Soil particle size influence experiment: Three contaminated soils were roughly ground and sieved through 10, 60, 100, and 200 mesh standard screens to collect different particle size fractions. Cr(VI) content in each fraction was determined to analyze particle size effects.

## 4. Conclusions

This study reveals that the soil at the chromium slag site in North China exhibits strong alkaline characteristics, with severe Cr(VI) pollution. Cr(VI) accounts for 13–22% of total chromium content, far exceeding the limit specified in China’s Soil Environmental Quality: Risk Control Standard for Soil Contamination of Development Land (GB 36600-2018) [7]. Among the factors influencing Cr(VI) transformation, acidic conditions and ferrous sulfate (FeSO_4_) addition emerge as the two most critical drivers of Cr(VI) reduction, whereas humidity and temperature variations exert weaker impacts compared to chemical interventions.

In practical remediation scenarios, adjusting soil pH to 4–6 (via acid regulators) can promote Cr(VI) speciation transformation and enhance reduction efficiency. Simultaneously, the addition of FeSO_4_ effectively reduces Cr(VI) concentrations, and combining this with a soil moisture content of 20–25% facilitates the diffusion of reducing agents and microbial metabolism, synergistically improving remediation efficiency.

Micro-mechanistic analyses further indicate that the influence of soil particle size on Cr(VI) is not determined by a single specific surface area effect but depends on mineral composition (e.g., iron–manganese oxide content). Additionally, the spatial co-distribution of Cr with redox-sensitive elements (e.g., iron and manganese) confirms the adsorption-reduction coupling effect at the “mineral-chromium” interface.

Based on these findings, future research should focus on three directions: First, extend the experimental duration for temperature and humidity studies to fully elucidate long-term microbial-mediated environmental effects. Second, integrate advanced techniques such as X-ray absorption fine structure (XAFS) to deeply characterize the binding forms of Cr(VI) with iron-manganese minerals. Third, develop efficient multi-factor synergistic remediation technologies to provide a scientific basis for the management of chromium-contaminated soils.

## Data Availability

The original contributions presented in this study are included in the article. Further inquiries can be directed to the corresponding author.

## Supplementary Materials

The following supporting information can be downloaded at: https://www.mdpi.com/article/10.3390/molecules30153076/s1, Table S1: Element and compound contents in soil samples; Table S2: Determination results of control groups and RMH-A043 certified reference material.
