# Supplementary material for: Influencing Factors of Hexavalent Chromium Speciation Transformation in Soil from a Northern China Chromium Slag Site
Source: Molecules. 2025 Jul 23;30(15):3076. doi: 10.3390/molecules30153076 (PMC12348942; doi:10.3390/molecules30153076)
Supplement: Supplementary file 1 [file molecules-30-03076-s001.zip › molecules-3751846-supplementary.pdf]

Supplementary Material A

Table S1. Element and compound contents in soil samples.

Element	Unit	BC-035	BC-036-3	BC-036-4-1
Na_2O	%	0.98	0.62	0.68
MgO	%	12.2	14.2	13.4
Al_2O_3	%	8.47	5.99	6.32
SiO_2	%	28.6	16.8	18.7
P_2O_5	%	0.11	0.07	0.08
SO_3	%	0.48	8.62	8.27
K2O	%	1.13	0.53	0.61
CaO	%	17.8	20.1	19.5
TiO_2	%	0.44	0.84	1.25
MnO	%	0.15	0.13	0.13
Fe_2O_3	%	7.54	7.64	9.47
TOC	%	2.05	2.96	2.66
Co	μg/g	106	96.8	91.8
V	μg/g	197	222	219
Cr2O3	μg/g	23087	24092	22399
Ni	μg/g	440	469	453
Cu	μg/g	25.3	24.5	28.4
Zn	μg/g	156	167	168
Ga	μg/g	12.5	11.3	13.1
As	μg/g	5.11	10.66	6.83
Rb	μg/g	43.9	20.8	24.8
Sr	μg/g	252	230	231
Y	μg/g	10.6	7.37	8.98
Zr	μg/g	99.5	112	167
Br	μg/g	4.92	5.62	6.26
Nb	μg/g	6.14	7.59	10.40
Ba	μg/g	815	636	691
Pb	μg/g	17.5	43.2	37.9

Table S2. Determination results of control groups and RMH-A043 certified reference material.

Treatment Group	BC-36-3 mg/kg	BC-36-4 mg/kg	BC-35 mg/kg	Certified Reference Material RMH-A043 155±12 mg/kg
PH=9	2063	1930	3488	147
Fulvic acid（0 mg/kg）	2200	1984	3542	156
Citric acid（0 mg/kg）	2168	1965	3518	146
$FeSO_4$（0 mg/kg）	2100	1883	3532	153
Moisture=2%	2184	1820	3325	157
Temperature=26°C	2136	1892	3516	156
Mean	2142	1912	3487	153
RSD (%)	2.46	3.14	2.34	3.17

Notes: In the control group, treatments marked with (0) indicate no addition of the corresponding substance, serving as baseline controls, while pH=9, soil moisture=2%, and temperature=26°C are specific condition control groups. These two types of controls form the experimental control system, providing a reference for analyzing the impacts of different treatment factors.

Combined with the analysis of RMH-A043 certified reference material, the accuracy assessment shows that relative errors of all treatment groups (including control, fulvic acid, and citric acid groups) range from -5.81% to 0.65%, indicating small deviations from the certified value and effective reflection of true sample contents. For precision, the relative standard deviations (RSD) of BC-36-3, BC-36-4, BC-35, and the certified reference material are 2.46%, 3.14%, 2.34%, and 3.17%, respectively, all below 5%, demonstrating good data repeatability. These results confirm the reliability and stability of the experimental data, providing strong support for subsequent analyses.
